# Genome-wide DNA methylation pattern in a mouse model reveals two novel genes associated with *Staphylococcus aureus* mastitis

**DOI:** 10.5713/ajas.18.0858

**Published:** 2019-04-15

**Authors:** Di Wang, Yiyuan Wei, Liangyu Shi, Muhammad Zahoor Khan, Lijun Fan, Yachun Wang, Ying Yu

**Affiliations:** 1 Key Laboratory of Agricultural Animal Genetics and Breeding, National Engineering Laboratory for Animal Breeding, College of Animal Science and Technology, China Agricultural University, Beijing 100193, China

**Keywords:** *Staphylococcus aureus*-infected Mouse Model, F-MSAP Method, DNA Methylation, Udder Tissue, Differentially Methylated Genes

## Abstract

**Objective:**

*Staphylococcus aureus* (*S. aureus*) is one of the major microorganisms responsible for subclinical mastitis in dairy cattle. The present study was designed with the aim to explore the DNA methylation patterns using the Fluorescence-labeled methylation-sensitive amplified polymorphism (F-MSAP) techniques in a *S. aureus*-infected mouse model.

**Methods:**

A total of 12 out-bred Institute of Cancer Research female mice ranging from 12 to 13 weeks-old were selected to construct a mastitis model. F-MSAP analysis was carried out to detect fluctuations of DNA methylation between control group and *S. aureus* mastitis group.

**Results:**

Visible changes were observed in white cell counts in milk, percentage of granulocytes, percentage of lymphocytes, CD4^+^/CD8^+^ ratio (CD4^+^/CD8^+^), and histopathology of mice pre- and post-challenge with *S. aureus*. These findings showed the suitability of the *S. aureus*-infected mouse model. A total of 369 fragments was amplified from udder tissue samples from the two groups (*S. aureus*-infected mastitis group and control group) using eight pairs of selective primers. Results indicated that the methylation level of mastitis mouse group was higher than that in the control group. In addition, NCK-associated protein 5 (*Nckap5*) and transposon *MTD* were identified to be differentially methylated through secondary polymerase chain reaction and sequencing in the mastitis group. These observations might play an important role in the development of *S. aureus* mastitis.

**Conclusion:**

Collectively, our study suggests that the methylation modification in *Nckap5* and transposon *MTD* might be considered as epigenetic markers in resistance to *S. aureus*-infected mastitis and provided a new insight into *S. aureus* mastitis research in dairy industry and public health.

## INTRODUCTION

Bovine mastitis is considered as a serious problem to the dairy industry [1,2], which reduces the quantity and quality of milk as well as threatens public health [3]. It was responsible for more than $2 billion annual losses to the US dairy sector [4] and about $35 billion losses to the world [5]. The incidence of subclinical mastitis in modern dairy farms is much higher than clinical mastitis (<5%) and normally around 26% to 68%. Subclinical mastitis can be detected by increased milk somatic cell counts (SCC) [6], 200,000 to 500,000 of cells/mL in milk was considered as an indicator of subclinical mastitis in dairy cattle, while SCC in healthy mammary gland is less than 100,000 of cells/mL. The main reason for increased SCC in mammary glands is the invasion of pathogens, nowadays, the major pathogen responsible for bovine subclinical mastitis is *Staphylococcus aureus* (*S. aureus*) [7].

Inflammation progress of udder tissue is controlled by genetic and epigenetic factors as well as pathogens. DNA methylation, one of the main epigenetic modification mechanisms, plays an important role in gene expression [8,9]. DNA methylation around the signal transducer and activator of transcription 5-binding enhancer in the casein alpha S1 promoter was shown to be associated with shutdown of αS1-casein synthesis during acute mastitis [10]. Our previous research revealed that bovine mastitis enhances the level of methylation in the promoter of CD4 molecule (*CD4*) and alternatively decreases the expression of *CD4* by blocking the transcription factor binding [11]. Chang et al [12] suggested toll-like receptor 4 promoter was linked to a recognized mechanism of epigenetic regulation of gene expression in *Escherichia coli* mastitis. In 2016, Song et al [13] found three genes (macrophage stimulating 1, neuregulin 1, and N-acetyltransferase 9) with DNA methylation changes can serve as potential biomarkers in *S. aureus* subclinical mastitis. These studies showed that epigenetics have a key role in bovine mastitis and should be evolved in the strategies of mastitis control. However bovine mammary gland tissue is difficult to obtain because of injury to animals and economic losses, thus searches for an animal model have been carried out [14].

The laboratory mouse is widely used for scientific research in epigenetics and disease progress. There is little information in the cited literature about whole genome DNA methylation changes in a *S. aureus*-induced mastitis mouse model. Methylation-sensitive amplified polymorphism (MSAP) analysis depends on two different DNA methylation-sensitive restriction isoschizomers (*Hpa* II and *Msp* I) for the same restriction site (CCGG) and has been extensively used to explore genomic DNA methylation levels and patterns because of its reliability, sensitivity and convenient operation [15]. Fluorescence-labeled MSAP (F-MSAP) which is based on fluorescently labeled primers is more sensitive, safer and effective than the original MSAP [16]. To determine the DNA methylation changeability in udder tissue of *S. aureus* mastitis, in the current study, genomic DNA methylation variation and related genes were investigated in a mouse model using the F-MSAP method.

## MATERIALS AND METHODS

### Care and use of animals

All protocols and procedures for the experimental mice were reviewed and approved by the Institutional Animal Care and Use Committee at China Agricultural University, China (Permission number: DK996). All the experiments were performed in strict accordance with the regulations and guidelines established by this committee. Before tissue sampling, the mice were euthanized by cervical dislocation. All efforts were made to minimize their suffering.

### Sample selection and size

A total of 12 out-bred Institute of Cancer Research female mice ranging from 12–13 weeks-old were selected to develop a mastitis model. *S. aureus* was collected from fresh milk of mastitis dairy cattle. Bacteriological culture of milk samples was performed according to National Mastitis Council standards [17]. A volume of 3 mL milk was mixed into trypticase soy broth containing 7.5% NaCl and cultured at 37°C for 18–24 h. The *S. aureus* was confirmed by specific halo and transparent ring around the colony. Thermonuclease (*nuc*) gene has been used for rapid identification of *S. aureus*, thus polymerase chain reaction (PCR) for amplification of *nuc* gene was performed to identify *S. aureus* to double check [18,19]. The primers of *nuc* gene are as follows: F: 5′-GCGA TTGATGGTGATACGGTT-3′, R: 5′-AGCCAAGCCTTGA CGAACTAAAGC-3′. A 50 μL of the *S. aureus* culture (5×10^6^ colony-forming unit) was carefully inoculated into the fourth abdominal pair teats (left and right) of the mammary gland of six mice with a blunt head capillary glass tube to induce *S. aureus* mastitis. Simultaneously, six healthy control mice (C1–C6) were inoculated with sterile, pyrogen-free saline.

### Physiological and biochemical indicators detection

#### Body temperature test

The temperature of the rectum of the mice was measured at 5 time points per day, i.e., 0 h, 6 h, 12 h, 18 h, and 24 h using an electronic thermometer.

#### Paraffin section and hematoxylin-eosin section

The 4th pair of udder tissue was collected after the mice were humanely killed through cervical dislocation. Then the tissues were paraffin embedded and sectioned, and finally hematoxylin-eosin (HE) staining was performed.

#### Milk white cell count

A total of 10 μL of milk was evenly spread onto the slides. Then after NEWMANS staining, the number of white cells in 10 fields was counted through microscope, and the average value was taken as the result.

#### Complete blood count

A total of 1 mL blood was collected from orbital sinus and complete blood count (CBC) (including granulocytes [GRN] and percentage of granulocytes [GRN %], lymphocytes [LYM] and percentage of lymphocytes [LYM %]) was conducted by Xiyuan Hospital CACMS, Haidian, Beijing, China.

#### *CD4^+^*/*CD8^+^* ratio

A total of 200 μL fresh peripheral blood was collected from orbital sinus, and the cell counts of T helper/inducer lymphocytes (CD4^+^) and T suppressor/cytotoxic lymphocytes (CD8^+^) were measured by flow cytometry (conducted by Xiyuan Hospital CACMS, China; antibody information: anti-CD4^+^CD8 antibody [EDU-2+733], Abcam, Shanghai, China). The CD4^+^/CD8^+^ ratio is the ratio of T helper cells (with the surface marker CD4) to cytotoxic T cells (with the surface marker CD8).

### Genomic DNA isolation from udder tissue

Related physiological and biochemical indicators as well as udder tissue samples were obtained at 24 h after intra-mammary infection (IMI) with *S. aureus*. The mice were euthanized using cervical dislocation method. The skin surface of the fourth pair of mammary glands was washed with 75% ethanol and dried; consequently, the udder tissues were sampled quickly and carefully. The collected udder tissues were used for DNA extraction with Wizard Genomic DNA Purification Kit (Promega, Madison, Madison, USA) and the genomic DNA was run in 1×Tris-boric acid-ethylene diamine tetraacetic acid on 1% agarose gel electrophoresis to check its integrity.

### DNA digestion, ligation and amplification

#### DNA digestion

DNA samples from both the mouse groups were digested with two different isoschizomer systems (*EcoR* I/*Hpa* II and *EcoR* I/*Msp* I), respectively, in a water bath at 37°C for overnight. The *EcoR* I/*Msp* I digestion system was performed in a reaction including 500 to 800 ng genomic DNA, 1 μL *EcoR* I, 1 μL *Msp* I, 4 μL 10×B4 buffer and 13 μL ddH_2_O. *EcoR* I/*Hpa* II digestion was performed in a reaction containing 1 μL genomic DNA, 1 μL *EcoR* I, 2 μL *Msp* I, 4 μL 10×B1 buffer and 12 μL deionized water.

#### Ligation

The ligation was then performed in a final volume of 30 μL including 12.5 μL enzyme-digested products, 1 μL (10 pmol) *EcoR* I adapter, 1 μL (10 pmol) *Hpa* II/*Msp* I adapters, 0.5 μL T4 ligase, 4 μL 10×T4 buffer and 9 μL ddH_2_O and incubated at 16°C overnight and then inactivated at 65°C for 10 minutes.

#### Pre-amplification polymerase chain reaction

Four microliter of the above ligation product was pre-amplified as a template in a final volume of 20 μL contained 42 ng H-M+1 primer, 41 ng E+1 primer, 0.1 μL Ex Taq polymerase, 1.6 μL dNTP, 1.2 μL MgCl_2_, 2 μL 10×Ex buffer and 9.5 μL deionized water. The PCR conditions were as follows: 94°C for 5 min; 30 cycles of 94°C for the 30 s, 56°C for 1 min, and 72°C for 1 min; and a final extension at 72°C for 7 min.

#### Selective amplification polymerase chain reaction

Selective amplification PCR was performed in a final volume of 20 μL including 4 μL pre-amplified products, 41 ng H-M+3 primer, 12 ng E+3 primer, 0.1 μL Ex Taq polymerase, 1.6 μL dNTP mixture, 1.2 μL MgCl2, 2 μL 10×Ex Taq buffer, and ddH_2_O. The PCR amplification reactions were performed using touch-down cycles under the following conditions: 94°C for 5 min; 13 touch-down cycles of 94°C for 30 s, 65°C (subsequently reduced each cycle by 0.7°C) for 30 s and 72°C for 15 s; 23 continued cycles of 94°C for 30 s, 56°C for 30 s and 72°C for 15 s; and extension at 72°C for 7 min. The adapters and primers used in the present study are summarized in Table 1.

### Silver staining

After the selective amplification PCR, the products were loaded into polyacrylamide gel electrophoresis (PAGE) and then silver staining was performed. The silver staining steps are as following.

Firstly, the PAGE gel was fixed in a solution of 75% ethanol for 15 to 30 min with gentle shaking. Then washed the gel three times using deionized water. Later, a total of 200 mL 0.5% AgNO_3_ solution was added, incubating the gel 30 min at room temperature with gentle shaking (100 to 120 rpm). After incubation, the AgNO_3_ solution was discarded and both sides of the gel were washed for 20 s using deionized water. Next, a total of 200 mL freshly made aqueous solution of 7.5% sodium carbonate was added and with gentle shaking for 5 min. Afterwards, a total of 1.25 mL solution of 40% formaldehyde was added, then the gel was incubated at room temperature with gentle shaking. After washing the gel carefully, stained bands appeared within a few minutes. The incubation continued until all desired bands appeared. Finally, the reaction was quenched by washing the gel in 1% acetic acid for a few minutes and then the gel washed several times with deionized water (10 min per wash).

### Fluorescence-labeled methylation-sensitive amplified polymorphism analysis

Based on the silver staining results, four pairs of selective-primers with more different bands were selected to be fluorescent labeled (FAM-labeled) to perform F-MSAP analysis.

**Figure 1:**
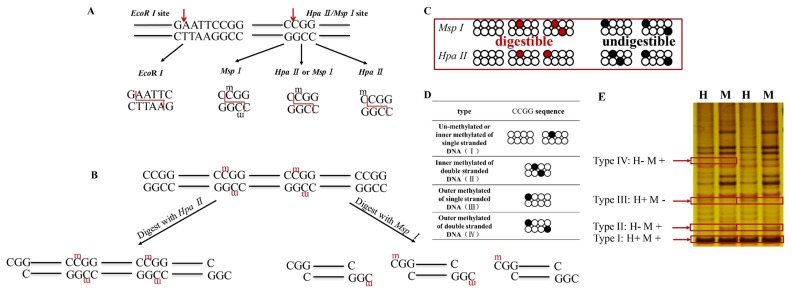
Schematic representation of the two isoschizomers and four DNA methylation patterns. (A) *Hpa* II is digestible in inner and outer methylated of single stranded CCGG suequece; *Msp* I is digestible in inner methylated of single and double stranded sequence; *EcoR* I is an internal control restriction enzyme which recognizes the GAATTC sequence. (B) An example DNA sequence is digested with isoschizomers (*Hpa* II and *Msp* I) and divided into different fragments. (C) Activity of the two isoschizomers. *Msp* I can recognize inner methylation of single and double stranded CCGG sequence, but cannot recognize outer methylation of single and double stranded CCGG sequence. *Hpa* II can recognize inner and outer methylation of single stranded CCGG sequence, but cannot recognize inner and outer methylation of double stranded CCGG sequence. The digested sites were shown in red circle and undigested sites are shown in black circle. (D) Illustration of four DNA methylation pattern types. (E) The agarose gel electrophoreses (silver stain) indicate four methylation types. Line M: a system of *EcoR* I/*Msp* I; line H: a system of *EcoR* I/*Hpa* II; H+: *Hpa* II digested; H-: *Hpa* II undigested; M+: *Msp* I digested; M-: *Msp* I undigested.

In the F-MSAP technique, the digestion of DNA was per formed with different methylation sensitive isoschizomers (Hpa II and *Msp* I) as well as an internal control restriction enzyme (*EcoR* I) as shown in (Figure 1A, 1B). The enzyme-digestion products were then ligated to adapters and pre-amplification and selective amplification with FAM-labeled primers was performed [16]. The amplified products with FAM-labeled primers were detected through the ABI3730 platform. Finally, the F-MSAP fragments were analyzed using GeneMarker V1.65 by detecting fluorescent signals of different intensity and locations. Four types of bands were detected. *t*-test and *chi*-square test were used in significance analysis of methylation levels between the two experimental groups. The level of statistical significance was set at p<0.05.

Ultimately, a total of 20 different bands were excised from polyacrylamide gels after silver staining, followed with secondary PCR and sequencing to identify the differentially methylated genes.

## RESULTS

### Physiological differences between *S. aureus* infected mice and healthy controls

**Figure 2:**
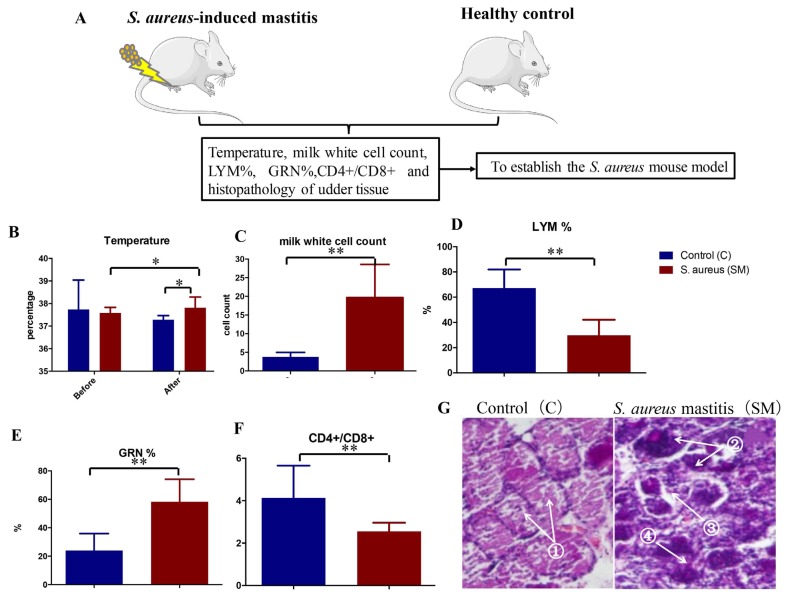
Differences of the physiological and biochemical indexes between the two groups. (A) Schematic sketch of establishing *Staphylococcus aureus* (*S. aureus*)-induced mastitis mouse model. (B) Temperature difference between healthy control and *S. aureus* mastitis; * p<0.05. (C) Milk white cell counts; ** p<0.01. (D) LYM %: percentage of lymphocytes; ** p<0.01. (E) GRN %: percentage of granulocytes; ** p<0.01. (F) CD4^+^/CD8^+^: CD4^+^/CD8^+^ ratio; ** p<0.01. (G) Udder tissue slices: ① Alveolus are complete in HC group, ② Inflammatory cells infiltration, ③ The space between the cells becoming wider, ④ Epithelial cell sloughing off can be found in SM group.

The *S. aureus* strain used to infect the mice was confirmed by specific PCR amplification of the *nuc* gene (Supplementary Figure S1). In order to get uniformity and standard *S. aureus*-infected mouse model, several parameters were checked pre- and post-challenge with *S. aureus* (Figure 2A).

Before the *S. aureus* attack, there was no difference for the body temperatures between C group and SM group (C: control group; SM: *S. aureus* induced mastitis group); however, a significant rise of body temperature was observed in SM group compared to the controls after 24 h IMI (Figure 2B). Furthermore, white cell counts in milk and GRN % were also increased (Figure 2C, 2E), while the LYM %, as well as CD4^+^/CD8^+^, were significantly decreased in SM group after IMI (Figure 2D, 2F). For further confirmation, paraffin sectioning and HE staining were carried out to check whether there was any abnormality in tissue, which showed inflammatory cells infiltration, the space between the cells becoming wider and with sloughing off of epithelial cells in SM group (Figure 2G). These findings indicated that the mouse model was launched successfully and provided a foundation for the subsequent studies (Figure 2).

### Four cleavage patterns of DNA methylation

The cleavage patterns were defined and shown in four types according to the methylation sensitivity of the isoschizomers as mentioned in Figure 1C and 1D. The PAGE gel electrophoresis indicated four methylation types shown in Figure 1E. Type I bands (un-methylated) appeared in both *EcoR* I/*Msp* I and *EcoR* I/*Hpa* II digestion. Type II bands (inner methylated of double stranded sequence) appeared only in *EcoR* I/*Msp* I digestion but not digested in the *EcoR* I/*Msp* I. Type III bands (semi-methylated, outer methylated of single stranded sequence) obtained in *EcoR* I/*Hpa* II digestion but could not be digested in *EcoR* I/*Msp* I. Type IV (outer methylated of double stranded sequence) represents the absence of bands in both enzyme combinations.

### Genome-wide DNA methylation profiles of the *S. aureus* infected mice and non-infected controls

**Figure 3:**
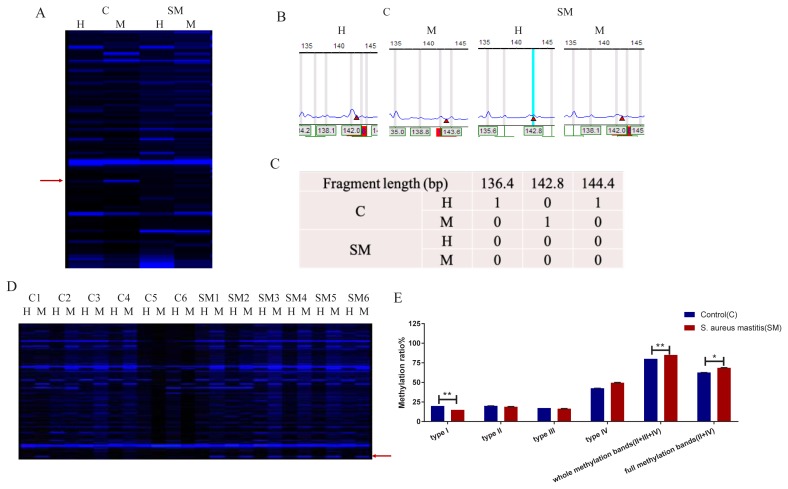
The electrophoresis and illustration of selective amplification. (A–C) An example showing how the signals in F-MSAP were named; (A) C: control group; SM: *Staphylococcus aureus* (*S. aureus*) induced mastitis group; Line M: a system of *EcoR* I/*Msp* I; line H: a system of *EcoR* I/*Hpa* II; (B) The signal peak represents methylation-sensitive amplified polymorphic fragments labeled with FAM fluorescent dye; the height of the peak represents the molecular weight of the fragments; (C) 1 = band, 0 = no band. (D) The fluorescent electrophoresis of the fourth primer of the 12 samples. C1–C6 are six replicates of control group; SM1-SM6 are six replicates of *S. aureus* induced mastitis group; Line M: a system of *EcoR* I/*Msp* I; line H: a system of *EcoR* I/*Hpa* II; red arrow indicated the different band pattern between the two groups. (E) DNA methylation levels of the four primers of the two groups. Whole methylation represents the sum of type II, type III, and type IV; full methylation represents the sum of type II and type IV.

Genome-wide DNA methylation profiles of the two groups were generated using the F-MSAP method. Eight pairs of selected primers were labeled with fluorescent dyes to detect DNA methylation patterns in udder tissue of the two groups. The fragments between 100 bp and 500 bp were highly intense. C1 (the first sample of C group) showed inner methylated of double stranded DNA and SM1 (the first sample of SM group) showed outer methylated of double stranded DNA (Figure 3A). The F-MSAP fragment gel files (Figure 3A) were captured by DNA sequencer and transferred into signal peaks (Figure 3B) and data (Figure 3C) through GeneMarker V1.65 software. A total of 369 clear bands were amplified from udder tissue samples of the two groups as shown in Figure 3D, and the bands in the red frame were the differentially methylated fragments.

### DNA methylation levels of the two groups

Methylation levels of the two groups were detected according to the bands of four FAM-labeled primers. The analysis of variance and Duncan’s multiple range tests were performed to evaluate the different methylation levels of the two groups. Variances of four DNA methylation patterns between two groups are shown in Supplementary Table S1. In the *S. aureus*-infected mouse group, the type I bands (un-methylated) are much lower than those in the control group (p<0.01). As for whole methylation bands (II+III+IV [%]) and full methylation bands (II+IV [%]), there were also significant differences between the two groups with p<0.01 and p<0.05, respectively (Figure 3E). These findings indicated that DNA methylation level in *S. aureus*-infected mice was significantly increased.

### *S. aureus*-induced changes in the differentially methylated bands in mouse udder tissue

All possible banding patterns between *S. aureus*-infected mastitis mice and healthy controls were explored to identify the changes in cytosine methylation patterns. Differentially methylated bands refer to the differences between the bands of M and H lanes in two experimental groups. The different DNA methylation patterns of the four FAM-labeled primers in the control group and SM group are shown in Supplementary Table S2. Out of 29 bands, three were highly significantly different, 25 bands showed significant differences and only one band remained unchanged under *S. aureus*-induced events between the two groups. These findings indicated there are frequent DNA methylation events when *S. aureus* attack udder tissue. The analysis of significantly differential methylated bands between two groups was carried out using *t*-test in EXCEL.

### Differentially methylated genes between *S. aureus*-induced mastitis mice and healthy controls

**Figure 4:**
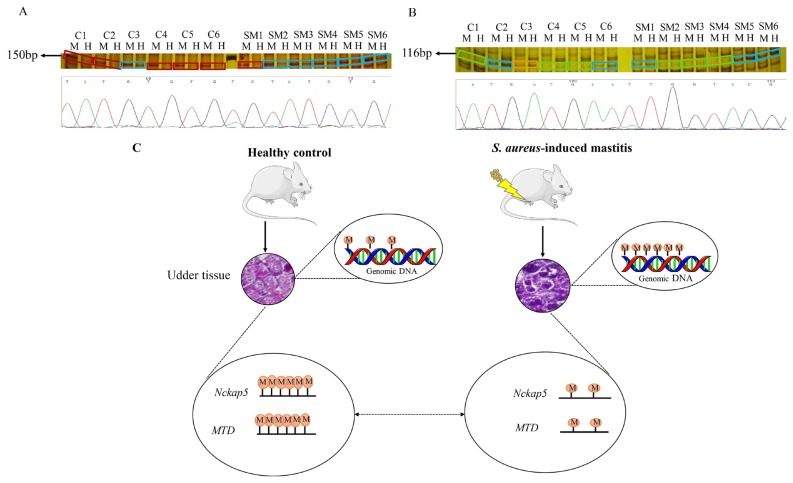
Silver staining and sequencing results of two differentially methylated fragments between SM mice group and controls. (A) Silver staining and partial sequencing result of gene NCK-associated protein 5 (*Nckap5*); C1–C6 are six replicates of control group; SM1-SM6 are six replicates of *Staphylococcus aureus* (*S. aureus*) induced mastitis group; Line M: a system of *EcoR* I/*Msp* I, line H: a system of *EcoR* I/*Hpa* II. Different color of the frames represents different DNA methylation pattern: light blue = Type I; wine red = Type II. (B) Silver staining and partial sequencing results of transposon *MTD*; C1–C6 are six replicates of control group; SM1-SM6 are six replicates of *S. aureus* induced mastitis group; Line M: a system of EcoR I/Msp I, line H: a system of *EcoR* I/*Hpa* II. Different color of the frames represents different DNA methylation pattern: light blue = Type I; orange = Type III; light green = Type IV. (C) The genome-wide DNA methylation level of *S. aureus*-induced mastitis mice is higher than that in healthy controls, gene *Nckap5* and transposon *MTD* showed hypo-methylation in *S. aureus* mastitis group. The circles with letter M inside indicates methylation, and the number of circles represents level of methylation.

To identify the differentially methylated genes, a total of 20 bands were excised from polyacrylamide gels after silver staining, followed secondary PCR and sequencing. Two differentially methylated fragments (100 to 200 bp) with different methylation patterns were confirmed as gene NCK-associated protein 5 (*Nckap5*) (150 bp) and transposon *MTD* (116 bp) through sequencing alignment (http://genome.ucsc.edu/cgi-bin/hgBlat) (Sequencing alignment results are shown in Supplementary Figure S2, S3). *Nckap5* is related to the promotion of the cell death [20], which should be methylated under normal conditions. In current results, *Nckap5* is hypo-methylated in *S. aureus* mastitis mice (Figure 4A). In SM group, except for SM1 showing type III (semi-methylated), other samples all showed type I (un-methylated); while in C group, except for C3 showed type I, others showed type II (inner methylated of double stranded sequence). Transposon *MTD* showed lower methylation level in *S. aureus* mastitis group compared with control group. In SM group, SM1, SM5, and SM6 showed type I (un-methylated) and SM2, SM3, and SM4 showed type IV (outer methylated of double stranded); while in C group, C1, C4, and C5 showed type IV (outer methylated of double stranded); C2 and C6 showed type I (un-methylated) and C3 showed type III (semi-methylated) (Figure 4B). Since *Nckap5* gene and transposon *MTD* were found to be hypo-methylated in *S. aureus* infected mouse udder tissue, we assumed that these two genes might trigger inflammatory changes in udder tissue (Figure 4C).

## DISCUSSION

The current study used a mouse model to evaluate the changes of genome-wide DNA methylation patterns and levels in udder tissues post *S. aureus* infection. Two differentially methylated genes (*Nckap5* and transposon *MTD*) were discovered which could be indicators of *S. aureus* mastitis.

Mice can serve as an appropriate research model for bovine mastitis study due to their low cost and ease of maintenance [21]. Here, a *S. aureus*-infected mouse model was established and optimized, which is the pre-requisite for obtaining reliable results. Fan et al [22] had evaluated the body temperature, udder tissue slices, milk white cell count, CBC and CD4^+^/CD8^+^ ratio pre- and post-challenge in order to establish the *S. aureus* mouse model, which is consistent with the present study. These observations suggest that our established *S. aureus* mouse model showed consistency and reliability for the further experimental procedure.

In the current study, we used F-MSAP method to detect genomic DNA methylation variance of udder tissues between *S. aureus*-induced mastitis mice and healthy mice. F-MSAP assay is modified from MSAP, which using fluorescently labeled primers and capillary gel electrophoresis instead of traditional denaturing acrylamide gel electrophoresis and silver staining [16,23,24]. Compared with MASP, F-MASP method has been proven to be safer, more efficient and with higher resolution because it can automatically detect genome-wide DNA methylation patterns with DNA sequencer. Based on F-MASP analysis, our findings revealed that the genomic DNA methylation level of mastitis mice was significantly higher than those in healthy mice. Similarly, a higher methylation level was found in the lymphocytes of *S. aureus* mastitis cows compared with healthy cows [13]. Thus, the data suggest that the methylation level of *S. aureus* mastitis-related genes may be changed because of DNA methylation fluctuations in *S. aureus* infected mice.

Subsequently, two differentially methylated genes ( *Nckap5* and transposon *MTD*) in mouse udder tissue were obtained, which may have important roles in the development or resistance of *S. aureus* mastitis. Our findings revealed hypo-methylation of *Nckap5* in *S. aureus*-induced mastitis mice. In a previous study, it was cited that human *NCKAP5* gene encodes Nck-associated protein 5 and the Nck protein reportedly plays a role in the process of the Fas ligand (FasL) factor-induced cell death [20]. These indicated that the *Nckap5* gene might have a role in promoting the death of immune cells. A genome-wide association study revealed that a polymorphism near *NCKAP5* showed significant link with symptoms of depression in humans [25]. A recent study reported that structural variations in cell line MHH-CALL-2 include homozygous inversion (*NCKAP5*) are responsible for disturbance of epigenetic gene regulation in childhood acute lymphoblastic leukemia by using next generation sequencing [26]. Moreover, in our results, we noted a higher level of methylation of transposon *MTD* in the healthy control group compared to the mastitis group, which is consistent with a recently reported study that showed the transposon is under a state of hyper-methylation in healthy status [27]. Transposon *MTD* belongs to Family *ERVL-MaLR* (mammalian apparent long terminal repeat [LTR] retrotransposons), which is mammalian apparent LTR-retrotransposons, which shows a lower expression in early embryos than other retrotransposons [28]. Studies suggested that DNA methylation status of the CACTA transposon can explain the incomplete penetrance and poor expressivity of the maize (*Zea mays*) mutant unstable factor for orange1 (*Ufo1*) [29]. Hypomethylation of *Karma* transposon is associated with the mantled phenotype in oil palm [30]. In aggregate, the data obtained in current research and the previously reported studies infer the importance of *Nckap5* and transposon *MTD* as indicators and they might be targets in remedies of mastitis. Further biological investigation is needed to validate the reliability of the current findings through pyro-sequencing and the gene expression via quantitative real-time PCR; in addition, the regulatory mechanisms of methylation changes could be study in mammary gland of dairy cow infected by *S. aureus*.

In conclusion, these findings offered a base line under standing of the investigation of dairy cow mastitis infected by *S. aureus* and provided a comprehensive picture of DNA methylation in *S. aureus* infected udder tissue. In addition, we suggested that the DNA methylation variation of *Nckap5* and transposon *MTD* might be considered as indicators and methylation markers in a control strategy of *S. aureus* mastitis, and provided a new insight into *S. aureus* mastitis research in dairy industry and public health.

## Supplementary Data











## Figures and Tables

**Table 1:** The adapters and primers used in Fluorescence-labeled methylation-sensitive amplified polymorphism

Name		5′→3′ Sequence
Adapters	*Hpa* II/*Msp* I adapters	GACGATGTCTAGAA
		CGTTCTAGACTCATC
	*EcoR* I adapters	CTCGTAGACTGCGTACC
		AATTGGTACGCAGTCTAC
Pre-selective primers	*Hpa* II/*Msp* I+T	GATGAGTCTAGAACGG-T
	*EcoR* I+A	GACTGCGTACCAATTC-A
Selective-primers	*Hpa* II/*Msp* I+TAC	GATGAGTCTAGAACGG-TAC
	*Hpa* II/*Msp* I+TAG	GATGAGTCTAGAACGG-TAG
	*EcoR* I+AAC	GACTGCGTACCAATTC-AAC
	*EcoR* I+ATG	GACTGCGTACCAATTC-ATG
	*EcoR* I+AAG	GACTGCGTACCAATTC-AAG
	*EcoR* I+ATC	GACTGCGTACCAATTC-ATC

Note: There are eight pairs of combined selective-primers as below:
Primer 1: F: GATGAGTCTAGAACGG-TAC; R: GACTGCGTACCAATTC-AAC Primer 2: F: GATGAGTCTAGAACGG-TAC; R: GACTGCGTACCAATTC-ATG Primer 3: F: GATGAGTCTAGAACGG-TAC; R: GACTGCGTACCAATTC-AAG Primer 4: F: GATGAGTCTAGAACGG-TAC; R: GACTGCGTACCAATTC-ATC Primer 5: F: GATGAGTCTAGAACGG-TAG; R: GACTGCGTACCAATTC-AAC Primer 6: F: GATGAGTCTAGAACGG-TAG; R: GACTGCGTACCAATTC-ATG Primer 7: F: GATGAGTCTAGAACGG-TAG; R: GACTGCGTACCAATTC-AAG Primer 8: F: GATGAGTCTAGAACGG-TAG; R: GACTGCGTACCAATTC-ATC
