# Supplementary material for: Genome-wide DNA methylation pattern in a mouse model reveals two novel genes associated with Staphylococcus aureus mastitis
Source: Asian-Australas J Anim Sci. 2019 Apr 15;33(2):203–11. doi: 10.5713/ajas.18.0858 (PMC6946959; doi:10.5713/ajas.18.0858)
Supplement: Supplementary file 1 [file ajas-18-0858-suppl1.pdf]

Table S1. The comprehensive differences in the four DNA methylation pattern between two groups of the four Selective-primers primers

NO.	Type of band				Sum of bands	Whole methylation bands	Fully methylation bands
	I（%）	II（%）	III（%）	IV（%）	I+II+III+IV	II+III+IV（%）	II+IV（%）
C1	59（15.99）	50（13.55）	76（20.60）	184（49.86）	369	310（84.01）	234（63.41）
C2	76（20.60）	72（19.51）	47（12.74）	174（47.15）	369	293（79.40）	246（66.67）
C3	79（21.41）	77（20.87）	77（20.87）	136（36.86）	369	290（78.59）	213（57.72）
C4	78（21.14）	82（22.22）	65（17.62）	144（39.02）	369	291（78.86）	226（61.25）
C5	66（17.89）	90（24.39）	54（14.63）	159（43.09）	369	303（82.11）	249（67.48）
C6	84（22.76）	74（20.05）	65（17.62）	146（39.57）	369	285（77.24）	220（59.62）
M±SD	73.67±9.31	74.17±13.48	64.00±13.48	157.17±18.72		295.33±9.31	231±14.33
（%）	（19.96±+0.03）	（20.10±0.04）	(17.34±0.03）	（42.59±0.05）		（80.04±0.03）	（62.69±0.04）
SM1	65（17.62）	110（29.81）	32（8.67）	162（43.90）	369	304（82.38）	272（73.71）
SM2	69（18.70）	71（19.24）	71（19.24）	158（42.82）	369	300（81.30）	229（62.06）
SM3	48（13.01）	84（22.76）	66（17.89）	171（46.34）	369	321（86.99）	255（69.11）
SM4	42（11.38）	43（11.65）	81（21.95）	203（55.01）	369	327（88.62）	246（66.67）
SM5	50（13.55）	52（14.09）	60（16.26）	207（56.10）	369	319（86.45）	259（70.19）
SM6	57（15.45）	59（15.99）	54（14.63）	199（53.93）	369	312（84.55）	258（69.92）
M±SD	55.17±10.42	69.83±24.38	60.67±16.82	183.33±22.10		313.83±10.42	253.17±14.50
（%）	（14.95±0.03）	（18.93±0.07）	（16.44±0.05）	（49.68±0.06）		（85.05±0.03）	（68.61±0.04）
p-value	0.009	0.659	0.700	0.051		0.009	0.025

Note: C1-C6, control mice; SM1-SM6, *S. aureus*-infected mice. P-values of four kinds of DNA methylation and proportion distribution were got from t-test and chi-square test, respectively.
