# Supplementary material for: Genome-wide DNA methylation pattern in a mouse model reveals two novel genes associated with Staphylococcus aureus mastitis
Source: Asian-Australas J Anim Sci. 2019 Apr 15;33(2):203–11. doi: 10.5713/ajas.18.0858 (PMC6946959; doi:10.5713/ajas.18.0858)
Supplement: Supplementary file 2 [file ajas-18-0858-suppl2.pdf]

Table S2. All discrepant bands in DNA methylation of the four Selective-primers primers

	bp	C1	C2	C3	C4	C5	C6	SM1	SM2	SM3	SM4	SM5	SM6	*p*-value
primer 4	74	3	4	4	4	4	3	2	1	2	3	3	3	0.007
	76	1	1	1	1	1	1	2	2	2	4	1	1	0.049
	79	4	3	4	4	4	3	1	1	2	3	3	4	0.033
	99	2	1	1	2	2	2	4	4	4	4	3	2	0.001
	236	2	2	2	2	2	2	2	4	2	4	4	2	0.049
	243	2	2	1	2	1	1	2	1	2	4	4	4	0.046
	254	3	4	3	3	3	3	4	3	4	4	4	4	0.018
	265	4	3	3	4	3	4	4	4	4	4	4	4	0.049
	294	2	2	2	2	2	1	2	2	2	4	4	4	0.035
	311	3	3	3	3	3	3	3	3	3	4	4	4	0.049
	328	1	1	1	1	1	1	2	1	1	4	4	4	0.022
primer 5	50	4	2	2	2	1	1	1	4	4	4	4	4	0.049
	65	4	2	2	2	2	2	4	4	4	4	4	4	0.001
	68	4	2	2	2	1	4	4	4	4	4	4	4	0.013
	101	4	1	1	1	1	1	2	3	3	3	3	4	0.024
	111	3	3	4	4	3	4	4	4	4	4	4	4	0.049
	126	3	3	2	1	1	1	2	3	3	3	3	4	0.035
	143	4	2	2	4	2	4	4	4	4	4	4	4	0.049
	150	4	2	2	2	2	2	4	2	4	4	4	4	0.018
	177	3	4	3	4	3	4	4	4	4	4	4	4	0.049
	202	4	2	2	2	2	2	2	4	4	4	4	4	0.018
	207	3	3	1	1	1	1	2	3	3	3	3	3	0.028
primer 6	55	3	4	3	3	4	3	4	4	4	4	4	4	0.010
	79	4	4	4	4	4	4	4	3	3	3	3	4	0.010
	87	1	1	3	4	4	3	1	1	1	1	1	1	0.014
	79	3	2	1	1	2	1	2	4	4	4	2	2	0.038
	165	4	4	4	4	4	4	4	4	4	4	2	2	0.145
primer 7	35	4	4	4	4	4	4	4	4	3	3	3	3	0.010
	117	4	2	2	2	4	4	2	2	2	2	2	1	0.035

Note: the number 1 represents the methylation pattern I, number 2 represents pattern II, number 3 represents pattern III, number 4 represents pattern IV.

Primer 4: F: GATGAGTCTAGAACGG-TAC; R: GACTGCGTACCAATTC-ATC

Primer 5: F: GATGAGTCTAGAACGG-TAG; R: GACTGCGTACCAATTC-AAC

Primer 6: F: GATGAGTCTAGAACGG-TAG; R: GACTGCGTACCAATTC-ATG

Primer 7: F: GATGAGTCTAGAACGG-TAG; R: GACTGCGTACCAATTC-AAG
