# Supplementary figures and images for: Genome-wide DNA methylation pattern in a mouse model reveals two novel genes associated with Staphylococcus aureus mastitis
Source: Asian-Australas J Anim Sci. 2019 Apr 15;33(2):203–11. doi: 10.5713/ajas.18.0858 (PMC6946959; doi:10.5713/ajas.18.0858)

Figure S1

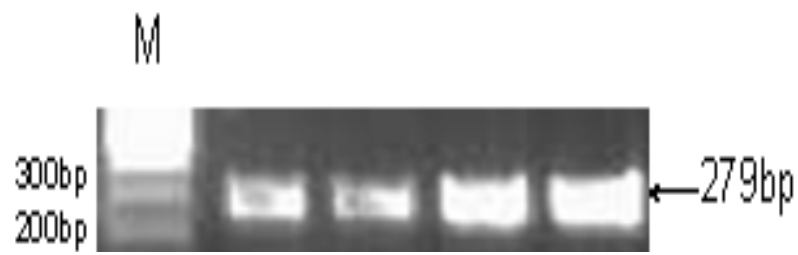

Figure S1. **PCR amplification of *nuc* gene.** M: marker I.

Supplement: Supplementary file 3 [file ajas-18-0858-suppl3.pdf]

Figure S2

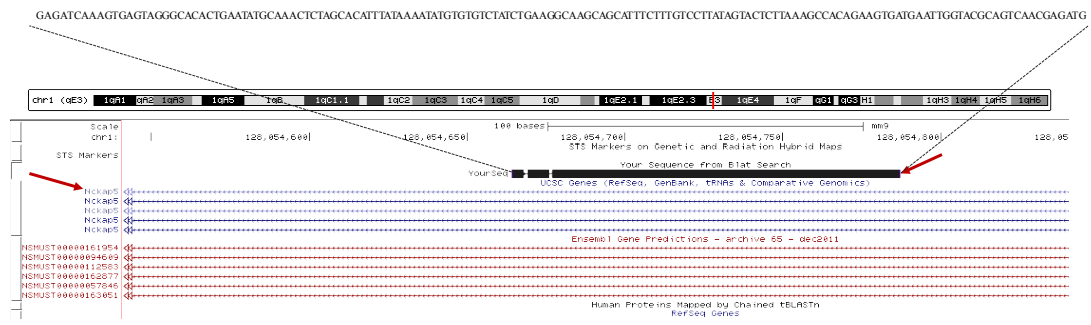

**Figure S2. Blast result of gene *Nckap5*.**

Supplement: Supplementary file 4 [file ajas-18-0858-suppl4.pdf]

Figure S3

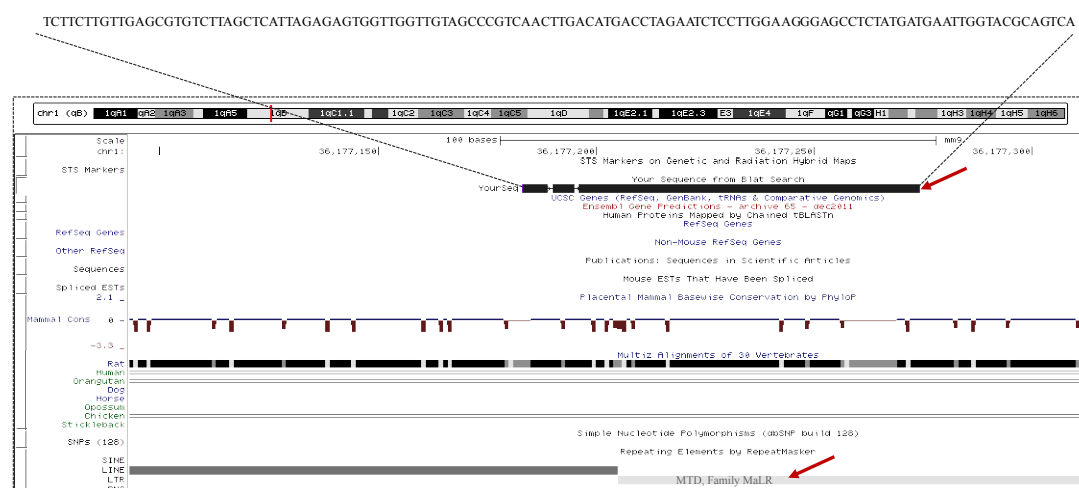

**Figure S3. Blast result of transposon *MTD*.**

Supplement: Supplementary file 5 [file ajas-18-0858-suppl5.pdf]
